# A Possible Case of Acquired Urea Cycle Disorder in a Critical Care Patient

**DOI:** 10.7759/cureus.92766

**Published:** 2025-09-20

**Authors:** Rabiu Momoh, Hamide Alijani, K S M Zibran Zalis Gaznavee, Elizabeth Ogundiya, Sara Sharp

**Affiliations:** 1 Critical Care Medicine, Medway Maritime Hospital, Gillingham, GBR; 2 Critical Care, Medway Maritime Hospital, Gillingham, GBR; 3 Critical Care Medicine, Medway NHS Foundation Trust, Gillingham, GBR; 4 Internal Medicine, Medway Maritime Hospital, Gillingham, GBR

**Keywords:** acute hyperammonemia, alcohol, critical care, genetic disorders, haemodiafiltration, haemodialysis, liver dysfunction, metabolic disorder, non-cirrhotic hyperammonemia, urea cycle disorder

## Abstract

Acute urea cycle disorder (UCD) presentation in patients can be challenging to manage, and the outcome can be deleterious if not promptly detected and urgently managed. This disorder is rare and can either be inherited or acquired. More descriptions of this disorder are noted in the paediatric population. We present a possible clinical scenario of acquired UCD in a middle-aged female with alcohol-related fatty liver disease who had a prolonged critical care admission, which culminated in an acute finding of a severe refractory acute hyperammonemia on her 67th day of critical care stay with a fatal outcome.

## Introduction

Urea cycle disorders (UCDs) are rare metabolic disorders caused by a lack of enzymes or transporters necessary for the urea cycle. This vital process in the liver transforms harmful ammonia, produced during protein metabolism, into urea for excretion by the kidneys. When this cycle malfunctions, it leads to hyperammonaemia, a potentially life-threatening condition that can result in severe brain damage or death if not treated promptly [1].

UCDs are uncommon; inherited forms are described to occur in around one in 35,000 live births. The most frequent type of inherited forms of UCD is ornithine transcarbamylase (OTC) deficiency, which is inherited in an X-linked pattern. This means that males are generally more severely impacted, although female carriers can also show symptoms, especially under metabolic stress. Other inheritable UCDs, such as carbamoyl phosphate synthetase I (CPS1) deficiency and citrullinemia type I, are inherited in an autosomal recessive manner [2].

Acquired cases of this disorder is increasingly gaining description in the literature. A comprehensive epidemiology of the acquired forms of UCDs is still less clearly defined due to its rarity and diverse causes. Acquired types arise later in life due to external factors that impair the function of the urea cycle, leading to hyperammonemia. Sepsis, liver conditions (e.g., cirrhosis, hepatitis, or acute liver failure), malignancies (affecting the liver or gastrointestinal system), certain medications (e.g., steroids, valproic acid), acquired mitochondrial disorders, prolonged fasting state, post-surgery (e.g., bariatric, post-liver transplantation), or inborn errors of metabolism unmasked by stress have been implicated as acquired causes of UCD. Clinical suspicion, biochemical testing, and genetic study evaluations are often needed in the assessment of UCDs. A heightened awareness of possible acquired causes of this potentially deleterious condition should be held among physicians. Elevated plasma ammonia with normal anion gap and glucose, an absence of known genetic mutations, and a possible history of triggering event would be useful diagnostic clues towards a consideration of a possible acquired UCD diagnosis. 

This case report aims to add further evidence to the burden of care that could result from a rare occurrence of an acquired UCD. Early detection and intensive treatment of symptomatic or acute UCD presentations in a critical care setting are essential to improving outcomes [3].

## Case presentation

We present the case of a 41-year-old female with a known history of alcohol excess and associated fatty liver disease, asthma and anxiety/depression who presented to the emergency department with a three-day history of visual hallucinations, reduced oral intake, and bilateral lower limb swelling.

Pertinent findings on admission were severe hyponatraemia (103 mmol/l ref range: 135-145 mmol/l) with a low serum and urine osmolalities and a low urinary sodium. She was managed for hypervolemic hyponatraemia and admitted to the high-dependency unit (HDU) for further care and monitoring. Fluid restriction and cautious correction of sodium not exceeding 10 mmol/l per day with optimisation of electrolyte derangements and supportive alcohol withdrawal management.

**Figure 1:**
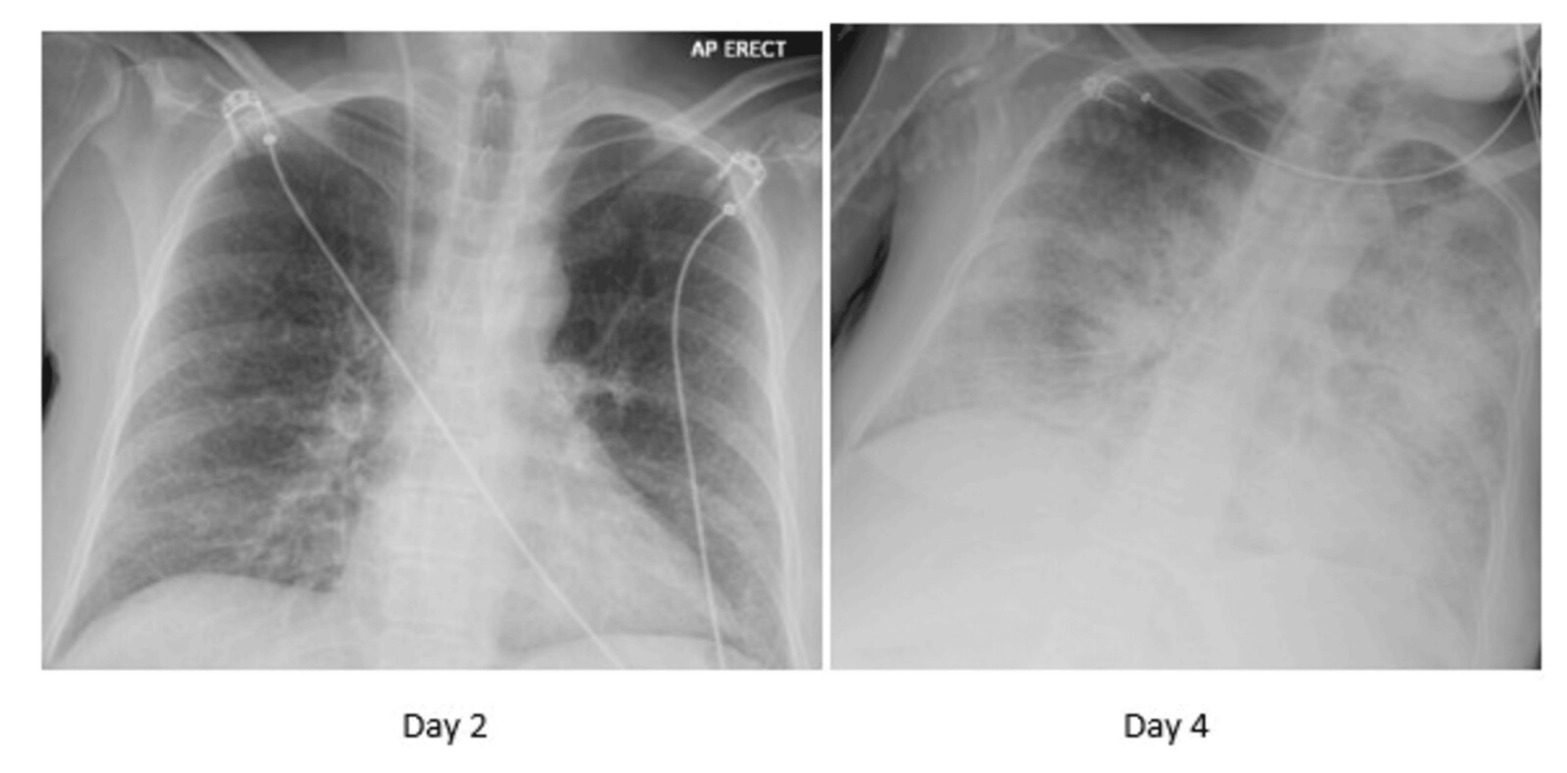
XR chest studies showing pulmonary congestion feature on an anteroposterior view XR chest study done on the patient on day 4

**Figure 2:**
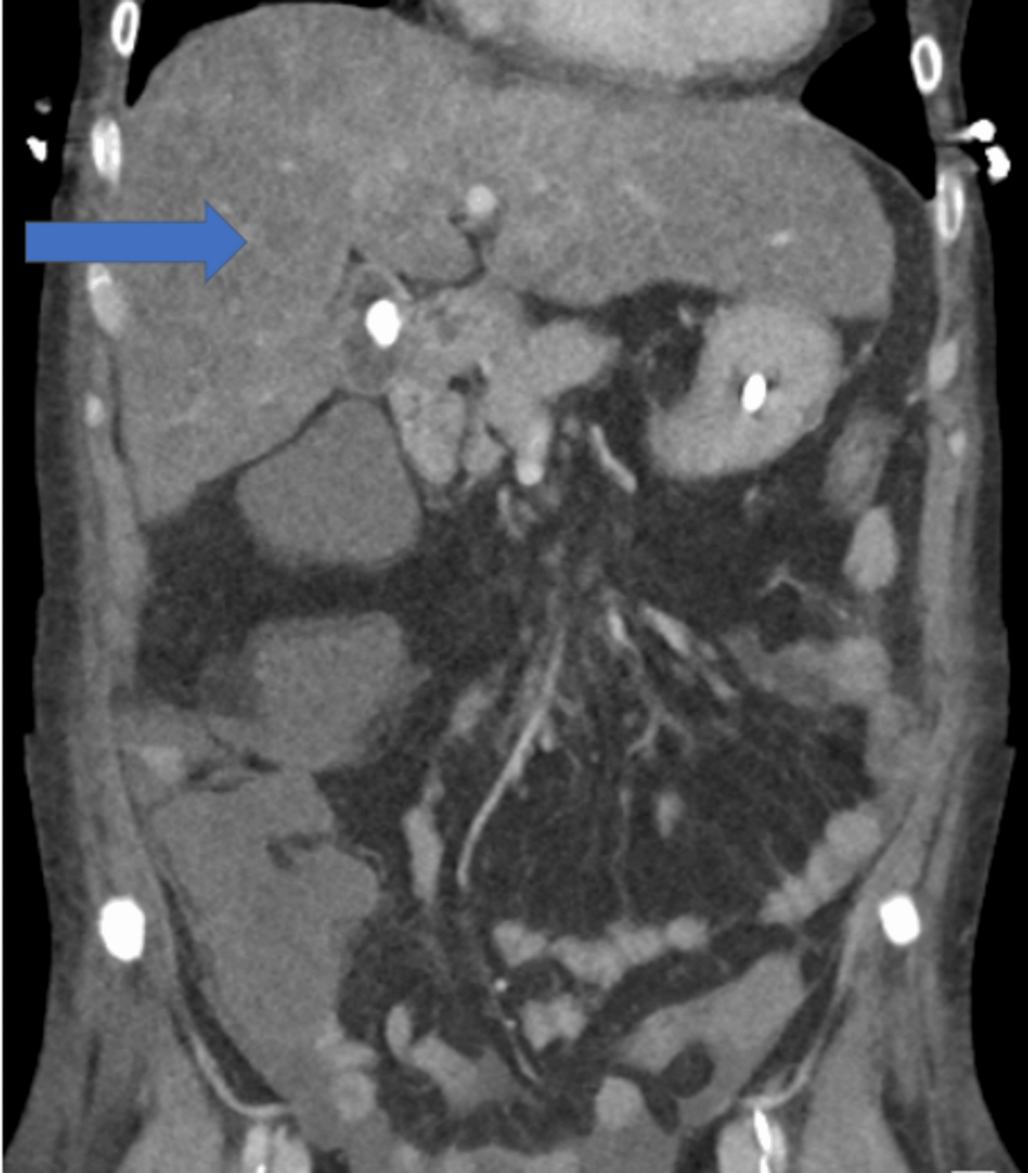
CT abdomen and pelvis (coronal section) undertaken on day 6 revealing the state of the liver (fatty liver changes with nodular margins)

Her HDU stay was complicated by the development of pulmonary congestion (see Figure 1) and subsequent respiratory failure requiring supplemental high-flow nasal oxygen administration on day 3. This worsened despite high-flow nasal oxygen and diuretics, and she required intubation on the intensive care unit (ICU) on day 5. A diagnosis of severe acute respiratory distress syndrome (with a PaO_2_ (7.9 kPa)/FiO_2_ (0.7) ratio of 11) and circulatory overload was made at this point. A contrast-enhanced CT study of her chest, abdomen, and pelvis (Figure 2) undertaken on day 6 revealing extensive bilateral lung consolidation with ground glass haziness and interstitial thickening, in-keeping with pulmonary oedema. She had moderate bilateral pleural effusions. A diffuse reduced attenuation of the liver was seen in keeping with hepatic steatosis. Hypertrophy of the caudate and left lobe of the liver was noted. The hepatic margins were nodular. No evidence of portal vein, splenic vein, and hepatic vein thrombosis was seen. Submucosal oedema in the large bowel, moderate ascites, and anasarca were noted on the CT report.

She was transferred on day 6 to a tertiary liver ICU for ongoing management. Due to delayed awakening off sedation on the ICU, she underwent an MRI brain study on Day 13 that showed changes consistent with minor osmotic demyelination syndrome (ODS) changes within the pons. Her ICU stay was also complicated by the development of oligo-anuric acute kidney injury requiring renal replacement therapy (RRT).

She was tracheostomized on day 14 of hospital admission (day 9 post-endotracheal intubation and initiation of mechanical ventilation) because of anticipated prolonged mechanical ventilatory support. During her stay on the liver ICU, she was diagnosed with aortic valve infective endocarditis-transthoracic echocardiogram (on day 21) showed a normal sized left ventricle with good systolic contraction but with moderate to severe aortic regurgitation. A trans-oesophageal echocardiogram (on day 24) revealed moderate-to-severe aortic regurgitation (probably from a small mass on the non-coronary cusp of the aortic valve). A positive blood culture result for a methicillin-sensitive *Staphylococcus aureus *(MSSA) was retrieved on day 25. A further multi-disciplinary meeting review of the trans-oesophageal echocardiogram imaging suggested the absence of vegetation on the aortic valve. She was recommended for surveillance as there were no urgent indications for surgery on the aortic valve and was managed medically with a planned six-week course of intravenous flucloxacillin. She had an ileus assessed on day 32 requiring total parenteral nutrition; re-initiation of enteral nutrition was restarted four days afterwards. She was diagnosed with a ventilator-associated pneumonia on day 47 requiring re-sedation and management. She was repatriated back to our district general hospital on day 52. Further supportive care was being facilitated on the ICU for recurrent delirium, physiotherapy unit-led rehabilitation, and tracheostomy weaning until day 58.

She developed new ventilator-acquired pneumonia (pseudomonas aeruginosa isolated in sputum) on day 59 associated with increased organ support requirements and re-sedation. This was managed supportively with antibiotics, she weaned off vasopressor support, and renal replacement therapy (continuous veno-venous hemodiafiltration) was briefly discontinued. On day 66, ongoing sedation requirement was reduced, but she failed to regain consciousness. As part of further evaluations, serum ammonia level was again requested, which was markedly raised at 802 µmol/l. Previously, serum ammonia levels were 32 µmol/l and 35 µmol/l on days 55 and 61, respectively. A recheck on the serum ammonia level on day 66 were 1507 µmol/l and >1800 µmol in the following few hours. This was associated with the development of refractory status epilepticus despite aggressive management with benzodiazepine, phenobarbital, levetiracetam, and lacosamide. She was treated with sodium benzoate, L-arginine, and L-carnitine in the case of a urea-cycle disorder alongside the other ammonia-lowering measures already initiated. Her liver function tests remained relatively normal at this point, and there was no ongoing exposure to medications like valproate or corticosteroids to account for the severe trigger of this severe hyperammonaemia.

**Figure 3:**
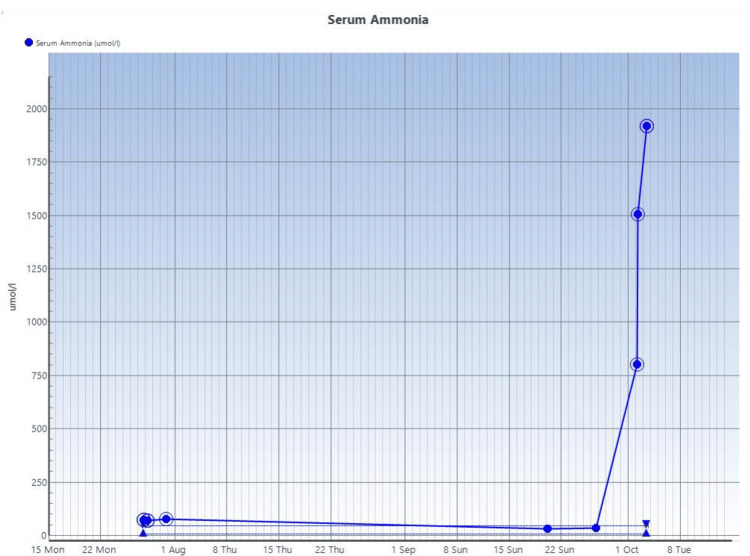
Serial ammonia studies done on the patient over the course of her admission

**Figure 4:**
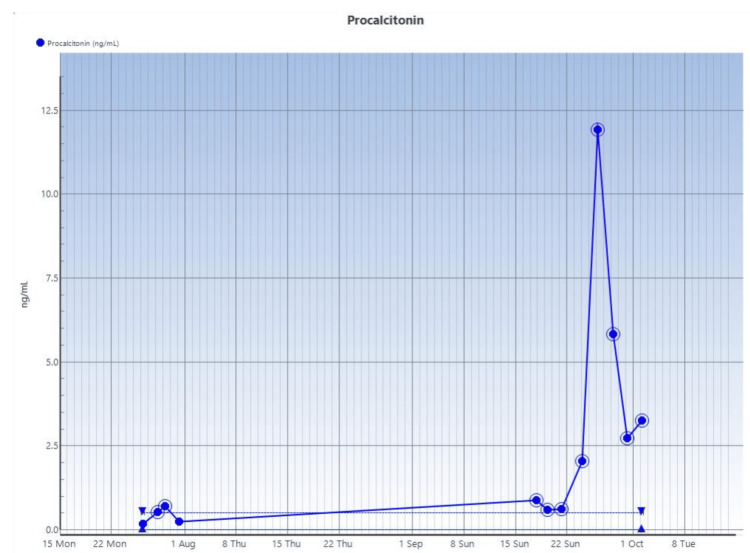
Serial procalcitonin studies done on the patient under review

**Figure 5:**
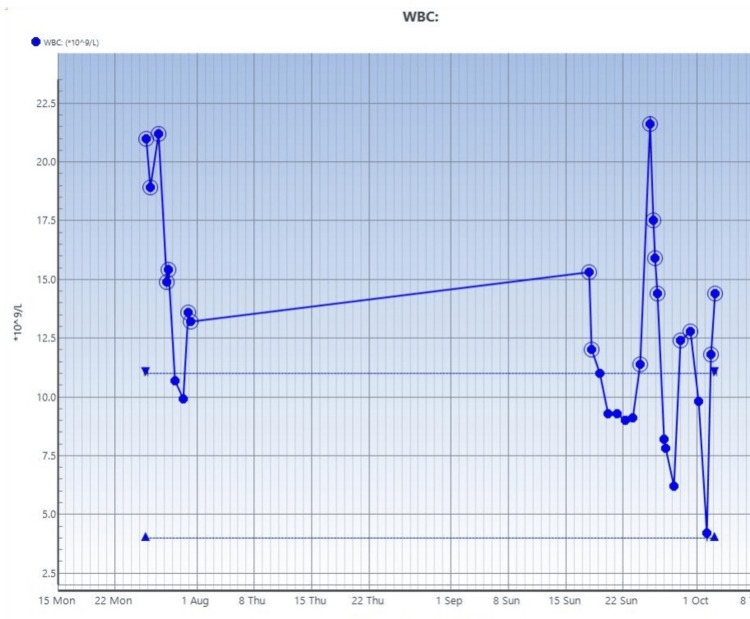
Trend of white blood cell counts on blood studies done on the patient

**Figure 6:**
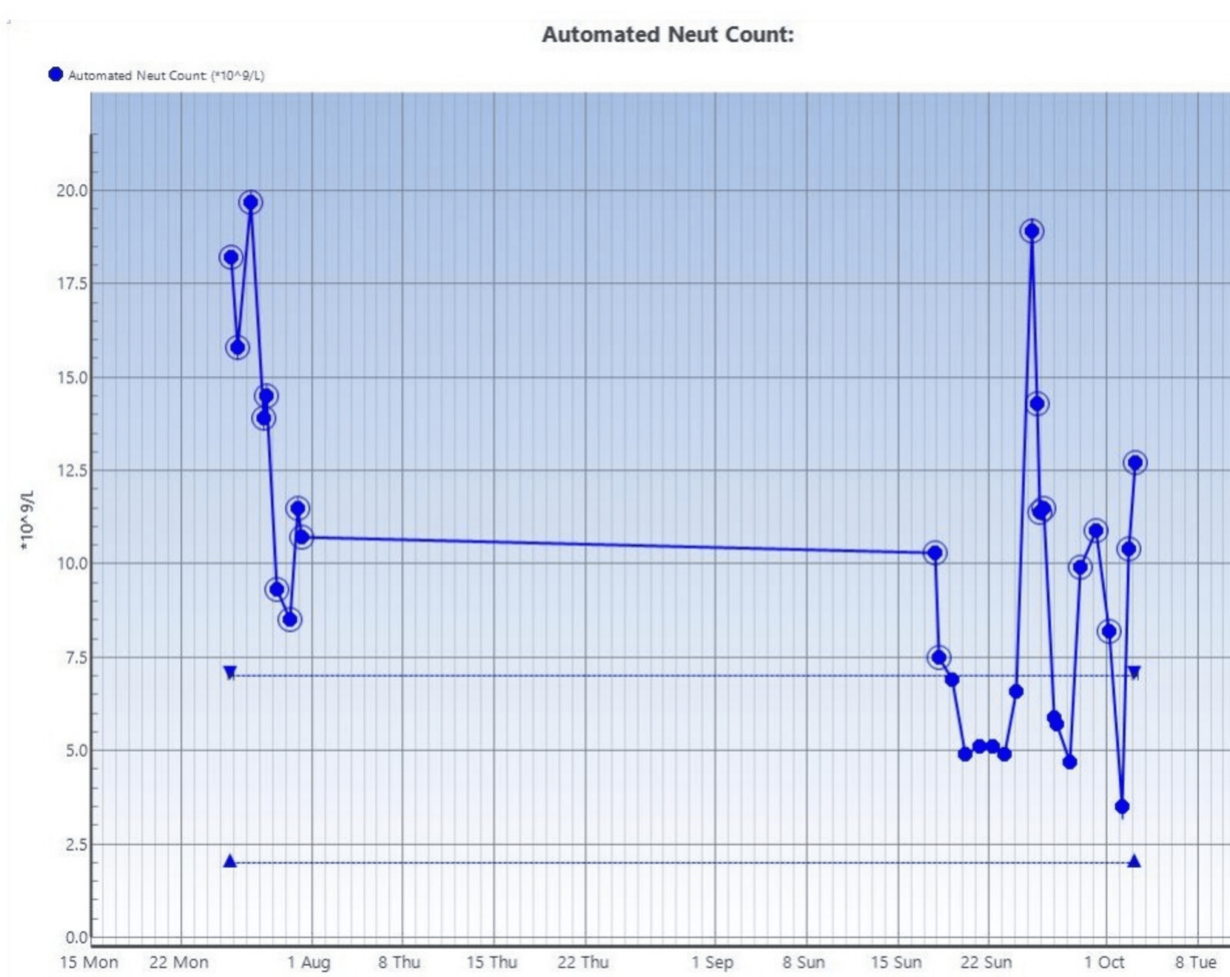
Serial neutrophil count on blood studies done on the patient

Multidisciplinary management of this patient's severe condition with acute refractory hyperammonaemia was facilitated with a metabolic disorder unit, hepatology unit, and neurology unit. Given the severity of hyperammonaemia and without a significant transaminitis, a presumptive diagnosis of a possible UCD was made, probably of acquired or an undiagnosed inherited causes. The results of the laboratory investigations ordered for this patient are shown in Tables 1-3 and Figures 3-6. 

**Table 1:** Results of other laboratory investigations undertaken on the patient

Test	Day 1 results	Day 68 results	Reference range
Serum phosphate	0.61		0.80-1.50 mmol/l
Serum alkaline phosphatase	269	133	30-130 U/L
Serum albumin	18	38	35-50 g/l
Serum adjusted calcium	2.22	2.47	2.20-2.60 mmol/l
Estimated GFR	>90	>90	>90 mL/min/1.73m^2^
Serum creatinine	30	50	45-84 umol/l
Serum sodium	103		133-146 mmol/l
Serum potassium	4.4		3.5-5.3 mmol/l
Serum C-reactive peptide	16.9		0.0-5.0 mg/l
Total bilirubin	118	77	0-21 umol/l
Serum alanine transferase	103	8	<35 U/l
Serum magnesium	0.50	0.98	0.70-1.00 mmol/l
Serum urea	<0.8	2.4	2.5-7.8 mmol/l
Serum procalcitonin	0.17	3.26	0.0 – 0.5 ng/ml
Serum hepatitis C antibody screen	negative		
Serum hepatitis B surface antigen screen	negative		
HIV 1-2 antibodies + p24 antigen screens	negative		
Anti-nuclear antibody screen	negative		
Alpha-1-antitrypsin	negative		
Alpha fetoprotein	3.9 KU/l		(< 7.4 KU/l)
Gastric parietal cell	Negative		
Anti-smooth muscle	Negative		
Mitochondrial Ab	Negative		
LKM antibody titre	Negative		
Anti-nuclear Ab	Negative		
Serum caeruloplasmin	0.18 g/l		0.20-0.60
Serum copper	8.8 umol/l		12.0-25.0

**Table 2:** Results of plasma studies done on day 67 of the patient's hospital admission

Test	Result	Units	Reference range	Comment
Plasma free carnitine	13.0	umol/L	15.0-53.0	Mildly low
Plasma serine	<25	µmol/L	60-172	Low
Plasma glutamate	350	µmol/L	24-214	High
Plasma alanine	1150	umol/L	112-686	Elevated
Plasma citrulline	39	umol/L	8-57	Within normal limits

**Table 3:** Microbiological investigation results

Blood culture	Day 25 sample: methicillin-sensitive *Staphylococcus aureus *
	Day 46 sample – no growth
	Day 59 sample – no growth
Urine cultures	Day 2, day 33 samples– no growth
	Day 60 catheter-stream urine sample: microscopy (cells/ml.), WBC: 1544, RBC: 686, epithelial: 40, culture: >10^5^ cfu/ml of - +++ yeast cells seen (*Candida* spp.)
	Day 62 catheter-stream urine: microscopy (cells/cumm): WBC: 18 cells/ml, RBC: 12 cells/ml, epithelial cells: 1 cell/ml. +++ yeast cells seen. Culture: 10^4^-10^5^ cfu/ml of *Candida* spp.
Sputum culture	Day 59 sample: *Pseudomonas aeruginosa*
Serum beta-D-glucan	Day 65: 37 pg/ml (ref: < 80 pg/ml) - comment: negative study
Serum *Aspergillus* antigen screen	Day 65: negative

Despite maximal supportive measures with renal replacement therapy, administration of L-ornithine L-aspartate (LOLA), L-carnitine, lactulose, and rifaximin, her serum ammonia remained extremely high (day 68: 1920 µmol/L). Her cardiovascular support with noradrenaline and vasopressin to achieve a mean arterial pressure at least 65 mmHg also worsened. 

On day 68, CT head (and venogram) study demonstrated diffuse cerebral oedema, loss of grey-white differentiation, and ventricular compression, suggesting severe global brain injury (Figure 2). Given a finding of fixed pupils and refractory multi-organ failure, the prognosis was deemed very poor and unsalvageable. Following family discussions, a withdrawal of active treatment on the ICU was done, and demise was the eventual outcome.

**Figure 7:**
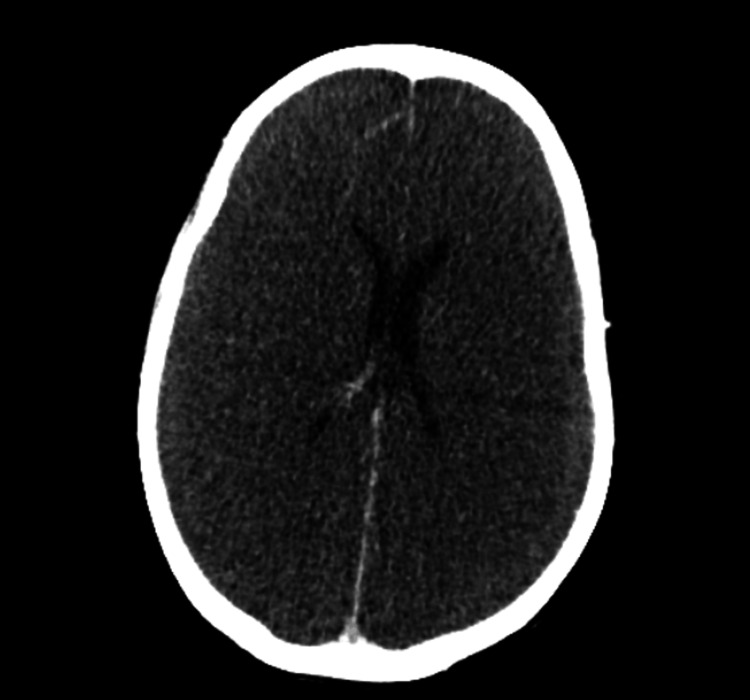
CT head study demonstrating diffuse cerebral oedema, loss of grey-white differentiation, and ventricular compression

## Discussion

The danger of hyperammonemia that results from the above UCDs is the untoward damaging effect on the brain, which could be irreversible if the hyperammonemia is not urgently addressed. Ammonia is osmotically active and readily crosses the blood-brain barrier. Within the brain cells, it is converted to glutamine intracellularly, which is also osmotically active. In acute accumulation, brain oedema results, and patients could manifest with symptoms ranging from mild to severe: confusion, vomiting, abnormal posturing, irritability, ataxic gait, hyperventilation (leading to respiratory alkalosis - making the cerebral oedema worse). In severe cases, seizures ensue - like in our patient's case. Patients could go comatose, and the risk of death becomes high. In patients with chronic UCD, they may manifest with behavioural disorders, anorexia, sleep problems, recurrent vomiting, generalized malaise, hallucinations, delusions, and psychosis. There may be no deranged hepatic enzymes found despite hyperammonemia in certain UCD patients. Other chronic states have been described to have fatty changes, cirrhosis, or even the development of hepatocellular carcinoma [4]. 

Plasma studies undertaken on our patient (as revealed in Table 1) include the presence of mildly low plasma free carnitine (at 13 umol/l) - plasma carnitine levels <5 umol/l are, however, usually suggestive of carnitine deficiency disorder, hence unlikely in our patient [5]. Our patient had a low plasma serine level on assessment; however, a further test for cerebrospinal fluid serine levels of 5-methyltetrahydrofolate and glycine, as well as a genetic study, would have been needed to exclude another important differential diagnosis named adult-onset serine deficiency disorder [6]. No family history of known genetic disorder was, however, reported by our patient’s family members.

Elevated plasma alanine and glutamate, as noted in our patient during her period with severe hyperammonemia, are biochemical consequences of disrupted nitrogen handling in hyperammonemic states, especially when peripheral tissues attempt to buffer excess ammonia [7]. Our patient's son and daughter underwent a genetic screen, and no evidence of chromosomal anomalies was found in them, raising a suspicion of an acquired cause of UCD in our patient.

UCD could manifest early in the neonatal period or could have a late onset in adolescence or adulthood. The critical care personnel could then be faced with the acute care of this rare disorder in the paediatric intensive care unit or the adult intensive care unit. Descriptions of acquired UCD are increasingly being noted in the literature, and in this case, hyperammonemia due to urea cycle enzyme deficiencies are noted without identifiable genetic disorders being found on genetic studies done. The use of tyrosine kinase inhibitors, nutritional deficiencies following bariatric surgery, and untreated fibrolamellar-type hepatocellular carcinoma have been implicated as possible causes of acquired UCDs, among others [8]. The acute precipitation of acute severe hyperammonemia in previously undiagnosed adult patients with UCD is increasingly being documented.

Anstey JR et al. (2015) described the precipitation of acute hyperammonemia in two patients (24- and 39-year-olds) following steroid administration for nasal septoplasty and chronic knee pain, respectively. Both patients went on to be diagnosed with ornithine transcarbamylase deficiency on genetic testing. Outcome for the first patient was a chronic morbid state with depressed sensorium at 22 months, and demise was the outcome in the second patient [9].

In terms of other precipitants, sodium valproate has been noted to possibly cause urea cycle enzyme dysfunction. Systemic illnesses, heavy protein load, surgery, and prolonged fasting states could be other potential causes of acute exacerbation of previously undiagnosed UCD states [10].

Surviving the impact of the acute hyperammonemia that can complicate UCD could be dependent on the time to assessment of this hyperammonemia and the effectiveness of treatment modalities deployed. Undertaking serum ammonia levels should be done where the cause of neurologic decline in patients cannot be easily explained. Where hyperammonemia is confirmed, care for this category of patient should be done in an intensive care unit, where, as needed, major organ support can be offered. The aims of treatment in severe acute hyperammonemic states would be to lower the ammonia level and reduce the potential debilitating impact on the brain. Early institution of hemodiafiltration or hemodialysis is suggested [11]. Ansten JR et al. (2015) suggested better gain in the use of intermittent hemodialysis over continuous hemodiafiltration [9]. Stopping the ongoing use of any offending agents or medications identified would be needed as well.

Multidisciplinary input (including the input from the metabolic disorder unit) should be obtained. The use of nitrogen scavengers (sodium benzoate, sodium phenylacetate), amino acid supplements (L-arginine hydrochloride), and N-carbamoyl glutamate has been suggested as initial therapies alongside hemodiafiltration or hemodialysis in this group of patients, pending when a specific UCD diagnosis is reached. Regular doses of rifaximin and lactulose should be prescribed. Considering limiting protein intake over the next 24-48 hours is suggested, but it is recommended to restart at lower amounts after this period. Carbohydrate and fat are suggested food classes in the acute period of the illness (first 48 hours). Further investigations with plasma and urine amino acid levels, plasma urea cycle enzymes assay, and genetic studies for UCD should be facilitated [9].

The role of liver transplantation in patients with UCD is noted in the literature. Patients with neonatal-onset UCD would often be considered for this intervention after the third month of life. Adult-onset UCD patients can be considered as well for this intervention [12]. Ikeri K et al. (2020) described the benefit of extracorporeal membrane oxygenation (ECMO) alongside other treatment measures (dialysis, nitrogen scavengers) in the treatment of an acute hyperammonemic occurrence in a neonate who ended up with an assessment of multienzyme UCD [13]. The literature describing ECMO use for similar scenarios among adults is scarce.

The role of hypothermic therapy (34-35 ℃ for 48 hours) for neuroprotection, liver cell therapy, stem/progenitor cell-based therapy, and gene therapy is an ongoing experimental therapy in the care of UCD [7]. From a public health point of view, more research into being able to identify undiagnosed adolescents and adults with underlying UCDs that risk exacerbation in an acute unwellness state is recommended. The critical care and medical personnel should be aware of this acute cause of neurologic deterioration to initiate timely care. 

## Conclusions

UCDs are life-threatening metabolic conditions that require a high degree of clinical suspicion in critically ill patients with hyperammonaemia. Early diagnosis, aggressive ammonia-lowering strategies, and multidisciplinary care are essential to improving survival and minimising long-term complications. Increased awareness among health personnel, coupled with advances in diagnostic and therapeutic approaches, will enhance outcomes for individuals with inherited or acquired forms of this rare disorder.

This case has been reviewed as a possible case of acquired UCD based on clinical, radiological, and available laboratory study findings on the patient. Our patient's children screened negative on genetic studies for inherited forms of UCD. The availability of genetic studies on the patient, the outcome of further plasma amino acid studies, and the urea cycle enzyme assay would have added more information towards confirming the type of UCD that the patient had.
